# Cerebral Creatine Deficiency Affects the Timing of Oligodendrocyte Myelination

**DOI:** 10.1523/JNEUROSCI.2120-21.2022

**Published:** 2023-02-15

**Authors:** Lauren M. Rosko, Tyler Gentile, Victoria N. Smith, Zeeba Manavi, George S. Melchor, Jingwen Hu, Nataliia V. Shults, Chris Albanese, Yichien Lee, Olga Rodriguez, Jeffrey K. Huang

**Affiliations:** ^1^Department of Biology, Georgetown University, Washington, DC 20057; ^2^Interdisciplinary Program in Neuroscience, Georgetown University, Washington, DC 20057; ^3^Department of Oncology, Georgetown University Medical Center, Washington, DC 20057

**Keywords:** cerebral creatine deficiency syndrome, creatine, guanidinoacetate methyltransferase, myelin, oligodendrocytes, remyelination

## Abstract

Cerebral creatine deficiency syndrome (CCDS) is an inborn error of metabolism characterized by intellectual delays, seizures, and autistic-like behavior. However, the role of endogenously synthesized creatine on CNS development and function remains poorly understood. Here, magnetic resonance spectroscopy of adult mouse brains from both sexes revealed creatine synthesis is dependent on the expression of the enzyme, guanidinoacetate methyltransferase (GAMT). To identify *Gamt*-expressed cells, and how *Gamt* affects postnatal CNS development, we generated a mouse line by knocking-in a GFP, which is expressed on excision of *Gamt*. We found that *Gamt* is expressed in mature oligodendrocytes during active myelination in the developing postnatal CNS. Homozygous deletion of *Gamt* resulted in significantly reduced mature oligodendrocytes and delayed myelination in the corpus callosum. Moreover, the absence of endogenous creatine resulted in altered AMPK signaling in the brain, reduced brain creatine kinase expression in cortical neurons, and signs of axonal damage. Experimental demyelination in mice after tamoxifen-induced conditional deletion of *Gamt* in oligodendrocyte lineage cells resulted in delayed maturation of oligodendrocytes and myelin coverage in lesions. Moreover, creatine and cyclocreatine supplementation can enhance remyelination after demyelination. Our results suggest endogenously synthesized creatine controls the bioenergetic demand required for the timely maturation of oligodendrocytes during postnatal CNS development, and that delayed myelination and altered CNS energetics through the disruption of creatine synthesis might contribute to conditions, such as CCDS.

**SIGNIFICANCE STATEMENT** Cerebral creatine deficiency syndrome is a rare disease of inborn errors in metabolism, which is characterized by intellectual delays, seizures, and autism-like behavior. We found that oligodendrocytes are the main source of endogenously synthesized creatine in the adult CNS, and the loss of endogenous creatine synthesis led to delayed myelination. Our study suggests impaired cerebral creatine synthesis affects the timing of myelination and may impact brain bioenergetics.

## Introduction

The creatine-phosphocreatine shuttle plays an essential role in energy metabolism (Wyss et al., 2007). During periods of high energetic demand, creatine kinases catalyze the transfer of the high-energy phosphate group in phosphocreatine to ADP, allowing for the rapid generation/regeneration of ATP, thereby maintaining the energetic supply required for cellular function (Wyss et al., 2007). About half of our daily creatine is derived from diet. The other half is endogenously synthesized by the conversion of glycine and arginine into guanidinoacetate through the enzyme arginine:glycine amidinotransferase (AGAT) and the subsequent transformation of guanidinoacetate into creatine through the enzyme guanidinoacetate methyltransferase (GAMT). Creatine can then be converted to phosphocreatine and used by cells endogenously or transferred to other cells through the creatine transporter, SLC6A8. Humans with mutations in *AGAT*, *GAMT*, or *SLC6A8* display cerebral creatine deficiency syndromes (CCDSs), which are rare diseases that are characterized by the disruption of the synthesis or transfer of creatine (Wyss and Kaddurah-Daouk, 2000; Braissant et al., 2011). If left untreated, children with CCDS can present with severe intellectual disabilities, seizures, developmental delays, autistic-like behaviors, and movement disorders, suggesting that the brain is particularly vulnerable to creatine deficiency (Giusti et al., 2019).

Previous studies suggest that two main waves of creatine synthesis occur during rodent CNS development: one from mitotic cells of the subventricular zone and the external layer of the cerebellum, and the second from oligodendrocytes starting at 2 weeks old and continuing into adulthood (Tachikawa et al., 2004, 2018; Baker et al., 2021). The profound increase in postnatal *Gamt* expression coincides with active CNS myelination (Sturrock, 1980; Baker et al., 2021), and suggests that oligodendrocytes may be a significant source of endogenous creatine within the postnatal brain. During active myelination, oligodendrocytes require a tremendous amount of energy for myelination. An estimated 3.24 × 10^23^ ATP molecules are required to synthesize 1 g of myelin (Harris and Attwell, 2012), and disruption of oligodendrocyte energetic metabolism (i.e., from hypoglycemia) causes a significant delay in CNS myelination (Yan and Rivkees, 2006; Rinholm et al., 2011). We have previously found that creatine protects oligodendrocytes from mitochondrial-mediated apoptosis during injury, and promotes remyelination (Chamberlain et al., 2017). However, whether endogenously synthesized creatine is necessary for developmental myelination or remyelination remains unclear. Since myelination is an energetically demanding process, we hypothesized that oligodendrocyte-derived creatine is required for developmental myelination and remyelination.

Here, we show that GAMT is the primary enzyme responsible for endogenously synthesized creatine in the mouse CNS. To track creatine production in the CNS, we developed a novel transgenic floxed mouse line that expresses GFP on the conditional excision of one or both copies of *Gamt*, to allow the tracking of cells which produce creatine, and for the analysis of *Gamt* loss of function in the mouse CNS, respectively. We found that oligodendrocytes are the main cells expressing GFP in the developing CNS, and that *Gamt* deletion resulted in delayed myelination and remyelination. Additionally, *Gamt* deletion may lead to altered neuronal bioenergetics in the adult brain. Our results indicate that oligodendrocytes are the major producers of creatine in the adult CNS, supporting previous observations (Tachikawa et al., 2018; Baker et al., 2021), and suggest that oligodendrocyte dysfunction through the loss of *Gamt* expression might contribute to conditions of creatine deficiency, such as CCDS.

## Materials and Methods

### Mice

All transgenic mice were maintained on a C57BL/6 background, and experiments were performed according to the protocol approved by the Institutional Animal Care and Use Committee at Georgetown University. Mice of both sexes were used for each experiment. *GAMT*^–/+^ mice for magnetic resonance spectroscopy (MRS) were a kind gift from Dirk Isbrandt (University of Cologne) (Schmidt et al., 2004) and back-crossed on C57BL/6 for four generations before breeding for MRS experiment. Floxed *Gamt (Gamt^fl/fl^*) line was engineered by Cyagen Biosciences where a linearized vector was electroporated into embryonic stem cells (C57BL/6). After confirming clones by Southern blotting, a chimera was produced by blastocyst microinjection. Floxed *Gamt* line was bred with Tg(CMV-cre)1Cgn (stock #006054) line from The Jackson Laboratory to first generate a heterozygous mutant (*Gamt*^*GFP*/+^). Heterozygous mutants were bred together to initially generate the KO mutant (*Gamt^GFP/GFP^*). For breeding experimental animals, female *Gamt*^*GFP*/+^ dams were bred with male *Gamt^GFP/GFP^* animals. Germline removal of *Gamt* allowed for removal of the excised allele without the passage of Cre transgene in our final experimental *Gamt*^*GFP*/+^ *and Gamt^GFP/GFP^* animals. Controls were *Gamt^fl/fl^*. Tg(Pdgfra-cre/ERT)467Dbe (stock #018280) from The Jackson Laboratory was used to generate inducible removal of *Gamt* from oligodendrocytes (OL *Gamt* iKO) with heterozygous Cre transgene. WT C57BL/6J (stock #000664) mice were also obtained from The Jackson Laboratory. Mice were maintained on a 12 h light/dark cycle with food and water *ad libitum.* All animals were fed a creatine-deficient amino acid diet (Crt def diet; Envigo; TD.01,084) unless otherwise specified (more details below).

### MRS

Animals underwent small animal imaging at the Preclinical Imaging Research Laboratory and the Center for Translational Imaging at Georgetown-Lombardi University Medical Center in a Bruker 7T/20 Magnetic Resonance Imager spectrometer incorporating Bruker AVANCE III electronics and ParaVision software version 5.1. Briefly, animals were anesthetized (1.5% isoflurane in a gas mixture of 30% oxygen and 70% nitrous oxide) and placed on a custom-manufactured (ASI Instruments) stereotaxic device with built-in temperature and cardiorespiratory monitoring engineered to fit a 25 mm Bruker mouse volume coil, as previously described (Fricke et al., 2006; Sirajuddin et al., 2012; Albanese et al., 2013). A T2-weighted two-dimensional anatomic locator scan was run followed by a volume-localized PRESS sequence with the following parameters: TE: 20 ms, TR: 2500 ms, averages: 1024, spectral width of 4 kHz, and 512,000 complex data points and 6 Hz line broadening, using a single voxel localized on the frontal cortex. All *in vivo* peak integrated areas were analyzed using Bruker's “TOPSPIN” software to assess relative differences in tissue chemistry, as described previously (Fricke et al., 2006; Sirajuddin et al., 2012; Albanese et al., 2013). The concentrations of metabolites were normalized to choline.

### Immunohistochemistry (IHC)

Mice were perfused with 4% PFA (Sigma) in PBS. Spinal cords and brains were removed and postfixed in PFA followed by 20% sucrose overnight. Brains were further cryoprotected in 30% sucrose before freezing in optimal cutting temperature medium (Sakura) on dry ice, then stored at −80°C. Twelve-micron sections of spinal cord or brain were sectioned on a cryostat and mounted on SuperFrostPlus slides. Slides were incubated in blocking solution (10% goat serum, 1% donkey serum, 0.25% Triton in TBS) for 1 h at room temperature. Mouse antibodies used an extra 1 h of mouse-on-mouse blocking reagent (Vector Laboratories). Primary and secondary antibodies were diluted in blocking solutions. Primary antibodies for IHC were as follows: chicken GFP (1:2000, Fisher Scientific catalog #PA1-86341, RRID:AB_931091), mouse GFAP (1:500, Sigma-Aldrich catalog #G6171, RRID:AB_1840893), rabbit Iba1 (1:1000, Fujifilm Wako Shibayagi catalog #019-19741, RRID:AB_839504), mouse NeuN (1:200, Millipore catalog #MAB377, RRID:AB_2298772), rabbit Olig2 (1:300, Millipore catalog #AB9610, RRID:AB_570666), mouse CC1 (1:200; Millipore catalog #OP80, RRID:AB_2057371), rat MBP (1:500, Millipore catalog #MAB386, RRID:AB_94975), mouse NKX2.2 (1:100, DSHB catalog #74.5A5, RRID:AB_531794), rabbit BCAS1 (1:1000, Synaptic Systems catalog #445003, RRID:AB_2864793), rabbit brain creatine kinase (BCK, 1:200, Abcam catalog #ab2117, RRID:AB_2080889), rabbit NG2 (1:500; Millipore catalog #AB5320, RRID:AB_91789), mouse Nestin (1:500, BD Biosciences catalog #611658, RRID:AB_399176), rabbit neurofilament (1:1000; Millipore catalog #N4142, RRID:AB_477272), and mouse nonphosphorylated neurofilament (1:1000; Millipore NE1023, RRID:AB_2043449). Antigen retrieval pretreatment was used for examining GFP expression. To use the 488 (FITC) channel in IHC without GFP interference, no antigen retrieval was used on slides.

### Western blot

Tissues were dissected from mice at various postnatal time points, flash frozen, and stored at −80°C. Tissues were digested in RIPA lysis buffer (Millipore), separated by SDS-PAGE, and immunoblotted with antibodies: guinea pig GAMT (1:500; Frontier Institute), rabbit AMPKɑ1 (1:1000; Abcam), rabbit p-AMPKɑ (1:1000; Cell Signaling), mouse myelin oligodendrocyte glycoprotein (MOG) (1:500; Santa Cruz Biotechnology), mouse nonphosphorylated neurofilament (1:5000; Cell Signaling), and rabbit β-actin (1:5000; Abcam). Proteins were detected using HRP-conjugated secondary antibodies and Pierce ECL Western blotting substrate. Membrane stripping was done with mild stripping solution (Fisher Scientific) and efficient stripping, or no signal, was confirmed by incubating with secondary antibody and reincubating with ECL.

### TUNEL

Slides were dried for 1 h before staining with Click-iT Plus TUNEL assay for *in situ* apoptosis detection (Fisher; C10618). Steps followed the manufacturer's instructions. A positive control slide was treated with DNase I.

### Tamoxifen

4-Hydroxytamoxifen (Sigma) was diluted in 100% ethanol and then diluted in peanut oil to a final concentration of 4 mg/ml. At 9-11 weeks of age, tamoxifen injections (1 mg) were administered intraperitoneally consecutively for 4 d ending 5 d before focal demyelination surgery.

### Spinal cord demyelination

Focal demyelination was induced by injection of 1.0% lysolecithin (Sigma) diluted in sterile PBS into ventral funiculus. Mice were killed for analysis at 5, 10, or 20 d after surgery.

### Cuprizone and special diets

All animals were fed a creatine-deficient amino acid diet (Envigo; TD.01084) unless otherwise specified. Demyelination was induced in 8-week-old male and female mice by adding 0.2% cuprizone (bis(cyclohexanone)oxaldihydrazone) into normal chow (LabDiet 5053) or creatine-deficient diet for 5 weeks. Cuprizone diet was replaced every 3 d to prevent stability concerns. All recovery diets used TD.01084 and added creatine or cyclocreatine (Sigma). Recovery diets after cuprizone were one of the following: normal chow, creatine-deficient diet, 2% creatine, or 0.1% cyclocreatine. Animals were weighed once every 2 d to ensure animals did not lose more than 10% body weight.

### Electron microscopy (EM)

Animals were perfused with EM fixation solution (4% PFA, 2% glutaraldehyde, 0.1 m sodium cacodylate buffer). Tissues were postfixed with 1% osmium tetroxide, and embedded in EmBed812. Ultrathin sections (70 nm) were poststained with uranyl acetate and lead citrate and examined in the Hitachi H7600 transmission electron microscope at 80 kV located at Georgetown University. Digital electron micrographs were recorded with the TIA software (FEI). Morphometric analysis was performed under blinded conditions by systematic uniform random sampling using 25 randomly selected images. ImageJ software (National Institutes of Health) was used to obtain axon diameter measurements from EM images taken at 5000× magnification and g-ratios using the freehand selection tool (>80 axons per animal; *n* = 1). For g-ratios, the inner myelin sheath diameter was divided by the outer myelin sheath diameter.

### Experimental design and statistical analyses

Images were collected on a Zeiss LSM 800 completed system confocal imager. Quantification of immunostaining was done by 1 or 2 blinded investigators using the ImageJ cell counter manually. For corpus callosum imaging, one medial and two lateral images of corpus callosum and cingulum were taken from 3 or 4 sections per slide (*n* = 3). For motor cortex imaging, a minimum of four images were analyzed from 3 animals. For spinal cord demyelinating lesions, the lesion was located by visualizing the accumulation of Hoechst-positive nuclei within the ventral white matter. A minimum of three lesion sections from 3 mice were analyzed for cell density. For cuprizone, three regions from four sections per animal were analyzed (*n* = 4). Density per square millimeter was calculated in Microsoft Excel as previously described by Chamberlain et al. (2017). All statistics were performed using Prism. Data are expressed as mean ± SEM. Comparisons were analyzed by two-way ANOVA with Sidak's multiple comparison test, one-way ANOVA with Tukey's multiple comparison test, or two-tailed *t* test. Diagrams were generated using Mind The Graph.

### Data availability

The datasets generated during and/or analyzed during the current study are available from the corresponding author on reasonable request.

## Results

### Cerebral creatine synthesis depends on *Gamt* expression

**Figure 1.**
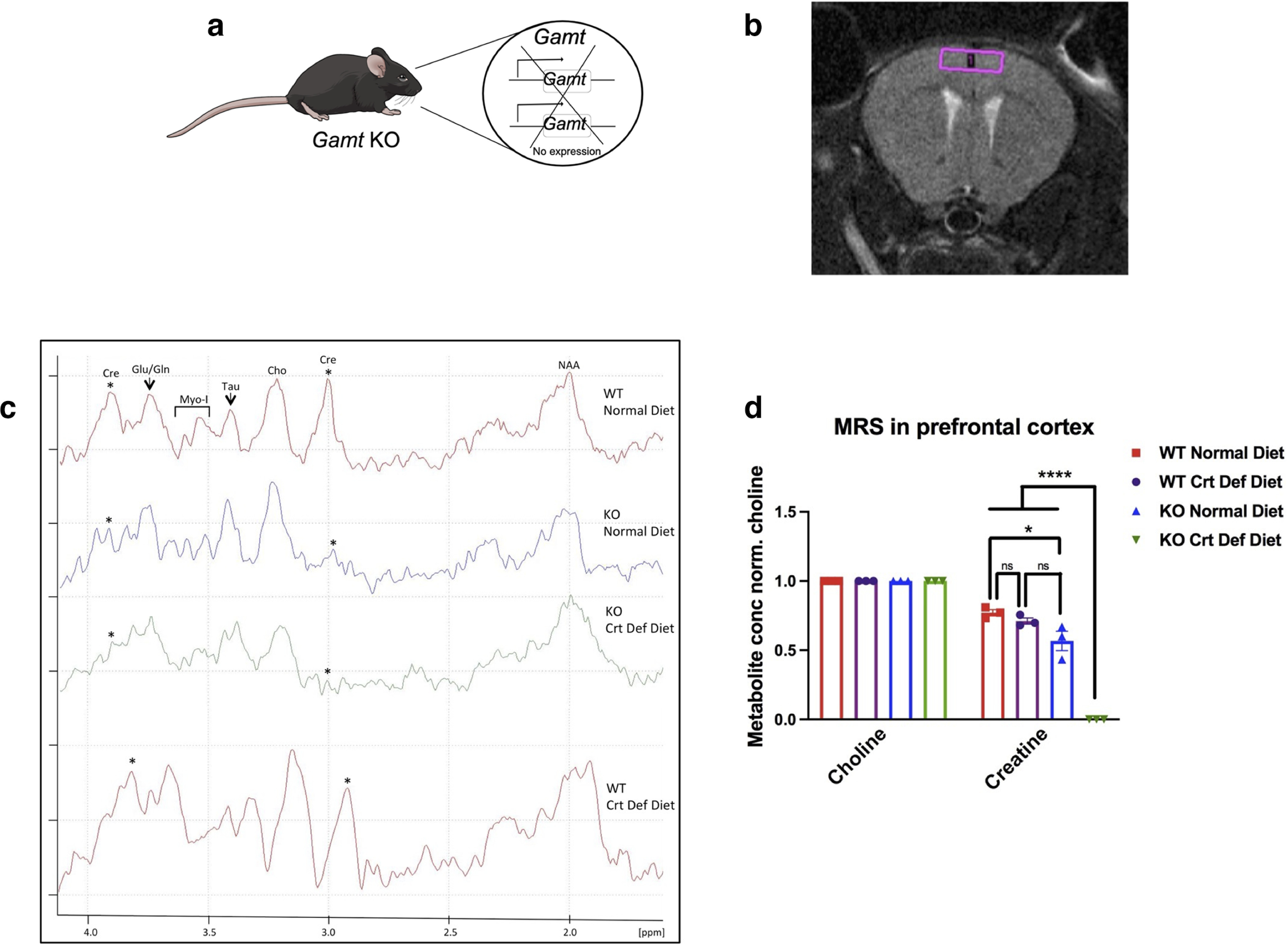
Endogenously synthesized creatine supplies cerebral creatine and is dependent on *Gamt* expression. ***a***, Diagram of *Gamt* KO transgenic mouse model. ***b***, Region of MRS voxel placement in mouse PFC. ***c***, Representative trace from MRS from each group. ***d***, Quantification of metabolites, normalized to choline, in KO on creatine-deficient diet (one-way ANOVA with Tukey's multiple comparisons; *F*_(3,8)_ = 83.81, df = 11, *p* < 0.0001) and in the WT on creatine-deficient diet (WT normal vs WT creatine-deficient *p* = 0.70, not significant). KO on a normal diet compared with WT (KO normal vs WT normal *p* = 0.02). Data are mean ± SEM; *n* = 3 biological replicates. *p < 0.05. **p < 0.01. ***p < 0.001. ****p < 0.0001.

Previous studies from our laboratory demonstrated that *Gamt* loss of function in mice impairs remyelination following experimental demyelinating injury (Chamberlain et al., 2017). However, these mice did not display obvious myelination defects or developmental abnormalities, suggesting that cerebral creatine levels may not have been disrupted during development. To determine whether cerebral creatine is detectable in the absence of *Gamt* expression, we performed ^1^H-MRS analysis in the PFC of 8-week-old *Gamt* KO (Fig. 1*a*) and WT mice (Fig. 1*b*). MRS is a specialized, noninvasive imaging-based technique that enables the metabolic profiling of tissues *in vivo*. The level of creatine was also compared with the levels of glutamate/glutamine, myo-inositol, taurine, choline, and N-acetylaspartate in the mouse PFC (Fig. 1*c*). We found that the KO mice on a standard rodent diet displayed measurable but significantly lower creatine levels in the brain compared with WT (Fig. 1*d*), suggesting that dietary creatine can partially compensate for cerebral creatine levels when GAMT is missing. MRS analysis was also performed on KO and WT mice on a creatine-deficient diet. We found that WT mice on creatine-deficient diet displayed similar creatine levels as those on standard diet. By contrast, *Gamt* KO mice on creatine-deficient diet displayed undetectable creatine levels in the PFC (Fig. 1*d*). These data suggest that endogenously synthesized creatine can supply adequate cerebral creatine in the absence of dietary creatine, and that GAMT is the main enzyme responsible for creatine synthesis in the mouse brain. Moreover, our data also suggest that dietary creatine can supply cerebral creatine in the absence of *Gamt* expression in mice, providing an explanation for the lack of obvious myelination impairment in our previous study (Chamberlain et al., 2017). To examine the effect of *Gamt* deletion on postnatal CNS development, all following studies were performed in mice on creatine-deficient diet.

### Oligodendrocytes are the major producers of creatine in the postnatal CNS

**Figure 2.**
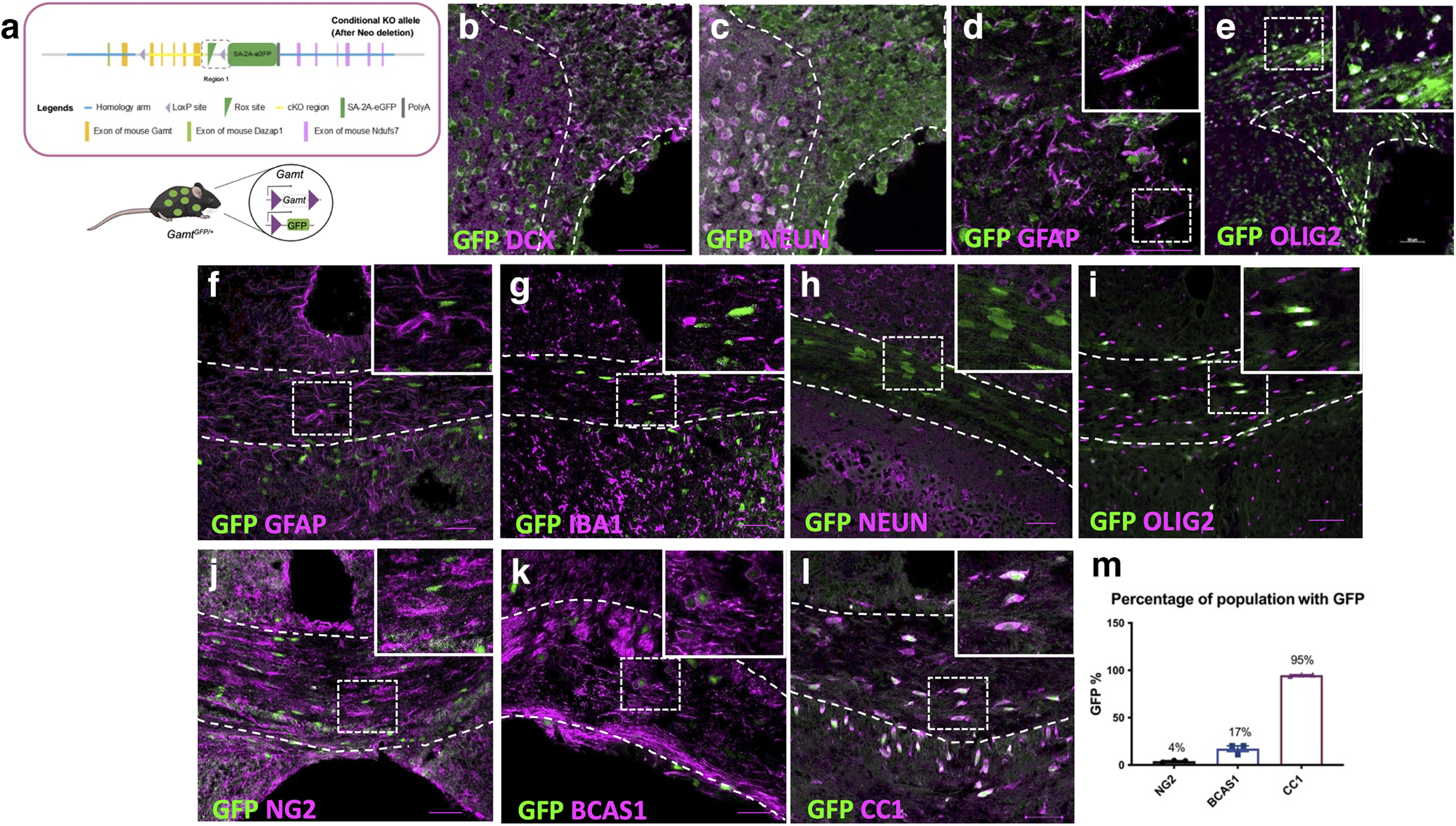
GFP is expressed in a variety of immature neurons and glia early in postnatal development but restricted to oligodendrocyte lineage cells during active myelination. ***a***, Diagram of transgenic mouse model and image showing heterozygous expression of *Gamt* with GFP expression with detailed outlined in Extended Data Figure 2-1. Analysis of GFP expression at P7 in (***b***) Doublecortin^+^ (DCX) cells in the subventricular zone (SVZ; outlined in white dotted line b-e), (***c***) neurons in adjacent striatum, (***d***) astrocytes, and (***e***) oligodendrocyte lineage cells in corpus callosum. Analysis of GFP expression at P14 in (***f***) GFAP^+^ astrocytes, (***g***) Iba1^+^ microglia/macrophages, and (***h***) NEUN^+^ neurons. Analysis of GFP expression in (***i***) oligodendrocyte lineage cells (OLIG2^+^), (***j***) NG2^+^ OPCs, (***k***) BCAS1^+^ early myelinating oligodendrocytes, and (***l***) mature CC1^+^ oligodendrocytes. ***m***, Percentage of OPCs, early myelinating and mature oligodendrocytes that express GFP. Data are mean ± SEM; *n* = 3 biological replicates. Scale bar: all images, 50 µm.

*Gamt* expression does not begin until very late in rodent embryogenesis and is regionally limited before birth (Braissant et al., 2005). As rodent development progresses, *Gamt* spatiotemporal expression changes drastically (Tachikawa et al., 2004; Braissant et al., 2005). To effectively track the expression of *Gamt* and identify the cell populations that synthesize creatine endogenously, we developed a transgenic mouse model that expresses a GFP reporter on the conditional excision of *Gamt*. This mouse line (*Gamt^fl/fl^*) contains LoxP sites flanking exons 2-6 of the *Gamt* gene (Fig. 2*a*; Extended Data Fig. 2-1). To delete *Gamt* developmentally and in all tissues, *Gamt^fl/fl^* were crossed with a cytomegalovirus Cre recombinase-expressing mouse line (*CMV-Cre*). Heterozygous mice containing one copy of *Gamt* (*Gamt*^*GFP*/+^) were used to track CNS cell populations that normally synthesize creatine while mice with homozygous deletion (*Gamt^GFP/GFP^*) were used for *Gamt* loss-of-function analysis. *Gamt^fl/fl^* mice without Cre recombinase were used as controls, and all animals were fed a creatine-deficient diet. IHC analysis for GFP coexpression in CNS cell types as indicators of endogenous creatine synthesis was performed on the cortical sections from postnatal (P) day P0 until P60.

10.1523/JNEUROSCI.2120-21.2022.f2-1 Figure 2-1 Overview of targeting strategy of transgenic mouse line. The line was engineered and generated by Cyagen biosciences A linearized vector was generated to the *Gamt* gene in C57BL/6 mice on chromosome ten and delivered to embryonic stem cells via electroporation. The targeted allele has loxP sites (purple) flanking exons 2-6 of the *Gamt* gene (yellow). The green Rox site prevents expression of enhanced GFP cassette but upon Cre recombination, *Gamt* along with the Rox site are removed and GFP is expressed. Download Figure 2-1, TIF file.

We observed low GFP expression in a variety of CNS cell types at P7, including neuroblasts (Fig. 2*b*), neurons (Fig. 2*c*), astrocytes (Fig. 2*d*), and oligodendrocyte lineage cells (Fig. 2*e*), which recapitulated previously published *Gamt* expression data (Tachikawa et al., 2018). However, by P14, when active developmental myelination is occurring in the CNS (Sturrock, 1980), this varied cellular expression was no longer observed, and GFP expression appeared to be restricted to distinctive cellular populations in the CNS. We found that GFP was not detected in GFAP^+^ astrocytes (Fig. 2*f*), IBA1^+^ microglia (Fig. 2*g*), or NEUN^+^ neurons (Fig. 2*h*), but was detected in OLIG2^+^ oligodendrocyte lineage cells in the corpus callosum (Fig. 2*i*). To further characterize endogenous creatine synthesis in oligodendrocyte lineage cells, immunostaining analysis for GFP expression and NG2^+^ oligodendrocyte precursor cells (OPCs), BCAS1^+^ early myelinating oligodendrocytes, and CC1^+^ mature oligodendrocytes was performed (Fig. 2*j–l*). We detected GFP expression in ∼4% of OPCs, 17% of early myelinating oligodendrocytes, and 95% of mature oligodendrocytes (Fig. 2*m*). These results support previous studies (Tachikawa et al., 2018; Baker et al., 2021), and suggest that endogenous creatine synthesis in the adult CNS occurs predominantly in mature oligodendrocytes during active myelination.

### *Gamt* deletion leads to reduced mature oligodendrocyte survival and delayed myelination

**Figure 3.**
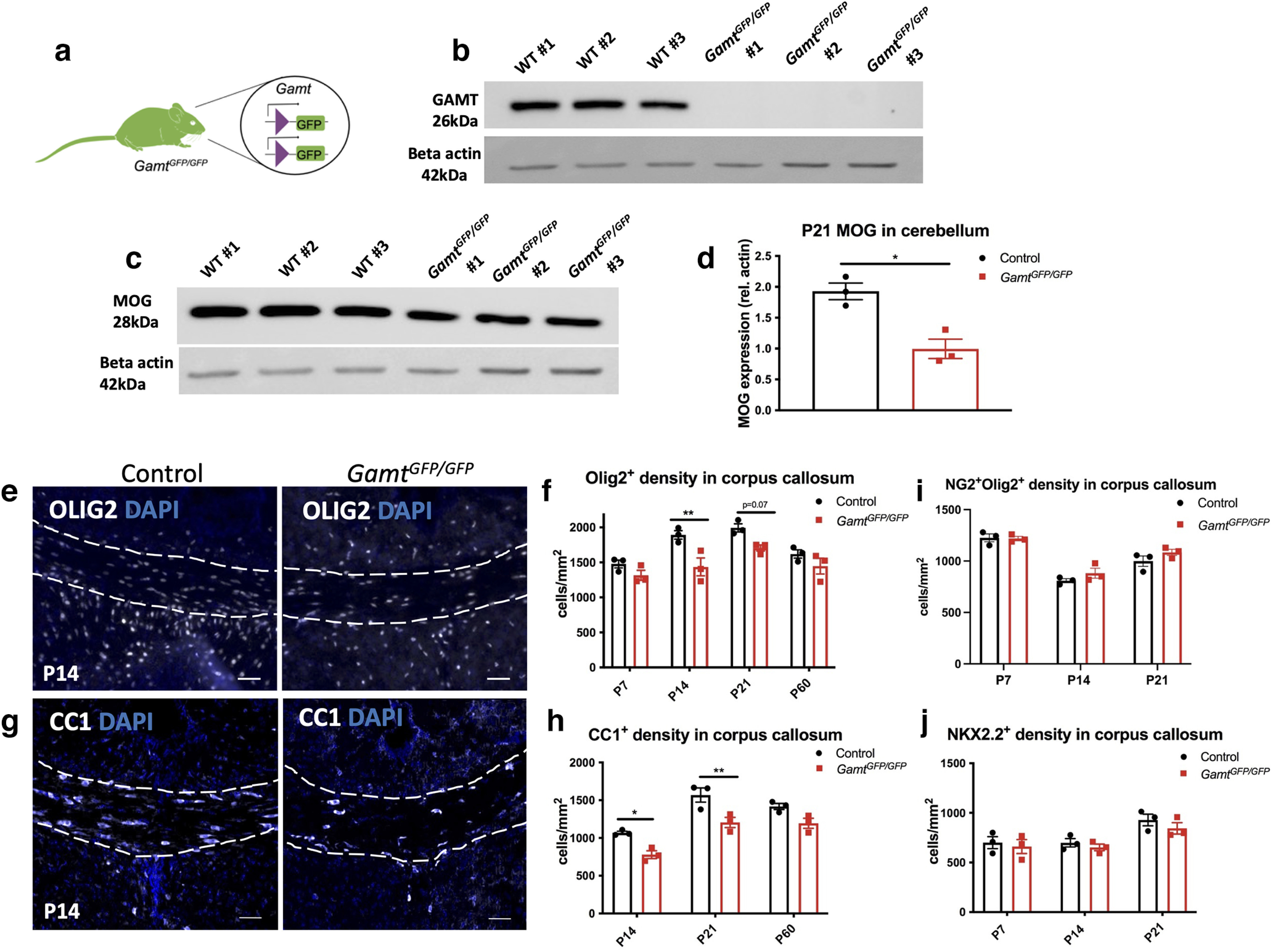
Deletion of *Gamt* leads to a reduction in mature oligodendrocytes at P14 and P21 and a delay in developmental myelination. ***a***, Visual representation of *Gamt^GFP/GFP^* transgenic mouse. Western blot analysis of P21 cerebellar lysate from *Gamt^GFP/GFP^* and *Gamt^fl/fl^* (WT control) for (***b***) GAMT and (***c***) MOG. ***d***, Quantification of MOG (two-tailed *t* test; *t* = 4.494, df = 4, *p* = 0.01). Immunostaining and quantification of (***e***,***f***) oligodendrocyte lineage cells at P14 (OLIG2^+^) (two-way ANOVA of genotype × age with Sidak's multiple comparisons; *F*_(1,4)_ = 19.77, df = 1 for genotype; *p* = 0.01), and (***g***,***h***) mature oligodendrocytes (CC1^+^) at P14 and P21 (two-way ANOVA with Sidak's multiple comparisons; *F*_(1,12)_ = 33.86, df = 1, *p* < 0.0001). Quantification of (***i***) NG2^+^ OPC and (***j***) NKX2.2 differentiating OPC density (*p* = 0.1, not significant) in the corpus callosum of *Gamt^GFP/GFP^* and control brain sections at P14. Data are mean ± SEM; *n* = 3 biological replicates. Scale bars: ***e***, ***g***, 50 µm. *p < 0.05. **p < 0.01.

We next determined whether *Gamt* loss of function affects CNS myelination in *Gamt^GFP/GFP^* mice (Fig. 3*a*). To confirm *Gamt* deletion, Western blot analysis for GAMT protein expression in mouse cerebellar extract was performed on *Gamt^GFP/GFP^* and control *Gamt^fl/fl^* mice at P21. The cerebellum was used to avoid any variability in tissue dissections between samples. We observed a complete loss of GAMT expression (26 kDa) in *Gamt^GFP/GFP^* mice compared with control, indicating that *Gamt* was effectively deleted in the *Gamt^GFP/GFP^* mouse CNS (Fig. 3*b*). We also observed a reduction of MOG expression in *Gamt^GFP/GFP^* mice compared with control, suggesting that GAMT loss of function affected the developmental myelination process (Fig. 3*c*,*d*). Next, immunostaining analysis was performed to examine the total number of oligodendrocyte lineage cells in the corpus callosum in *Gamt^GFP/GFP^* and control mice at P14 (Fig. 3*e*) and P21. We found that *Gamt^GFP/GFP^* mice exhibited significantly reduced OLIG2^+^ oligodendrocyte lineage cell number compared with control at P14 (Fig. 3*f*). A significant reduction of CC1^+^ mature oligodendrocytes in *Gamt^GFP/GFP^* mice was also observed at P14 and P21 (Fig. 3*h*). However, no significant changes in the number of OPCs labeled with either NG2 or NKX2.2 were detected in *Gamt^GFP/GFP^* mice compared with control at P7, P14, or P21 (Fig. 3*i*,*j*).

**Figure 4.**
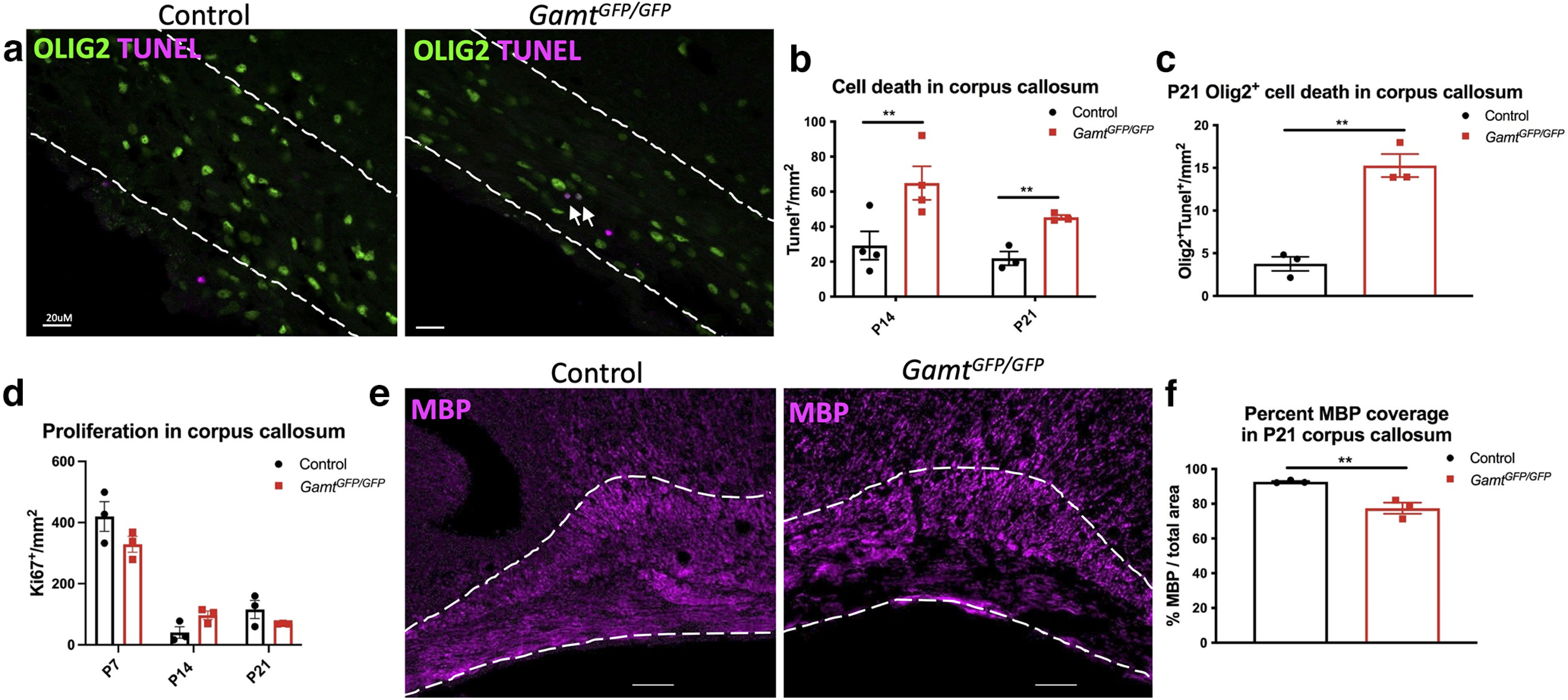
Deletion of *Gamt* leads to increased cell death of oligodendrocyte lineage cells and no difference in OPC proliferation in corpus callosum. ***a***, Images of TUNEL staining represent cell death density in the *Gamt^GFP/GFP^* animals at P14 and P21 in corpus callosum. Extended Data Figure 4-1*a* confirms cell death with caspase-3 staining. ***b***, Quantification of TUNEL labeled cells at P14 and P21 (two-way ANOVA with Sidak's multiple comparisons; *F*_(1,10)_ = 15.18, df = 1, *p* = 0.003), and (***c***) TUNEL^+^ oligodendrocyte lineage cells (TUNEL^+^OLIG2^+^) at P21 (two-tailed *t* test; *t* = 7.28, df = 4, *p* = 0.002). Extended Data Figure 4-1*b*, *c* shows TUNEL^+^ cellular colocalization with early myelinating oligodendrocytes. ***d***, Quantification of cell proliferation at P7, P14, and P21 (two-way ANOVA with Sidak's multiple comparisons; *F*_(1,8)_ = 0.06, *p* = 0.8134, not significant). Quantification of OPC proliferation in Extended Data Figure 4-1*d*, *e*. ***e***, MBP staining in corpus callosum (outlined) of *Gamt^GFP/GFP^* and control at P21. ***f***, Quantification of MBP percent coverage in the corpus callosum of *Gamt^GFP/GFP^* and control animals (two-tailed *t* test; *t* = 4.721, df = 4, *p* = 0.0092). Extended Data Figure 4-1*f*, *g* displays FASN in *Gamt^GFP/GFP^* animals. Data are mean ± SEM; *n* = 3 or 4 biological replicates. Scale bars: ***a***, 20 µm; ***e***, 50 µm. *p < 0.05. **p < 0.01.

To determine whether the reduction in mature oligodendrocytes was driven by decreased proliferation or increased cell death, we performed IHC at P14 and P21. First, we conducted a TUNEL assay for apoptosis, and observed increased TUNEL staining in the corpus callosum in *Gamt^GFP/GFP^* compared with control at P14 and P21 (Fig. 4*a*,*b*). This finding was also confirmed using caspase-3 staining (Extended Data Fig. 4-1). We observed a slight but significant increase in TUNEL labeling at the corpus callosum, and most of the dying cells were OLIG2^+^ oligodendrocyte lineage cells (Fig. 4*c*). Moreover, TUNEL-positive cells did not colocalize with markers of OPCs or mature oligodendrocytes in the *Gamt^GFP/GFP^* mice, but appeared colocalized with BCAS1, a marker of early myelinating oligodendrocytes (Fard et al., 2017) (Extended Data Fig. 4-1). To determine whether GAMT loss of function affected cell proliferation, we stained for Ki67, and found no significant difference in Ki67 expression in the corpus callosum between *Gamt^GFP/GFP^* and control mice at P7, P14, or P21 (Fig. 4*d*). Furthermore, we analyzed proliferation of OPCs (KI67^+^NG2^+^OLIG2^+^ cells) at P7 and observed no difference between groups (Extended Data Fig. 4-1). These results suggest that endogenously synthesized creatine does not regulate OPC proliferation in the developing corpus callosum but may be involved in oligodendrocyte maturation.

10.1523/JNEUROSCI.2120-21.2022.f4-1 Figure 4-1 Removal of GAMT leads to increased cell death and reduced fatty acid synthase in the corpus callosum. **a**) Quantification of caspase-3^+^ cell density at P14 (two-tailed t-test; t = 7.81, df = 4, p = 0.0015). **b**) Images of BCAS1^+^TUNEL^+^ dying early myelinating cells. **c**) Inserts showing colocalization of BCAS1+TUNEL+ in c. **d**) Quantification of OPC proliferation at P7 (two-tailed t-test; t = 2.005, p = 0.1155). **e**) Images of P7 OPC proliferation. **f**) Images of FASN^+^CC1^+^ cells in the corpus callosum at P21. **g**) Quantification of FASN^+^CC1^+^ cells (two-tailed t-test; t = 5.88, df = 4, p = 0.0042). Data are mean ± SEM with n = 3 biological replicates. Scare bar is 50µm in b and d. **p < 0.01. Download Figure 4-1, TIF file.

Since myelination depends on lipogenesis, we also examined the expression of fatty acid synthase (FASN) in the corpus callosum (Dimas et al., 2019). We observed complete colocalization between FASN and mature oligodendrocyte marker, CC1, and unsurprisingly, decreased FASN-labeled cells in *Gamt^GFP/GFP^* mice compared with control, suggesting reduced oligodendrocyte lipogenesis in the corpus callosum (Extended Data Fig. 4-1). To determine whether the lack of *Gamt* expression in mice affects myelination, immunostaining analysis for MBP was performed. We found a significant decrease in MBP coverage in the corpus callosum at P21 (Fig. 4*e*,*f*). However, we found that the overall number of oligodendrocytes and extent of myelination were similar between *Gamt^GFP/GFP^* and control mice by P60 (Fig. 3*h*). These results suggest that the rate of myelination is slower in the absence of creatine but eventually catches up to controls in the adult CNS.

### *Gamt* deletion leads to altered energetics and reduced BCK in neurons in the cortex of adult mice

**Figure 5.**
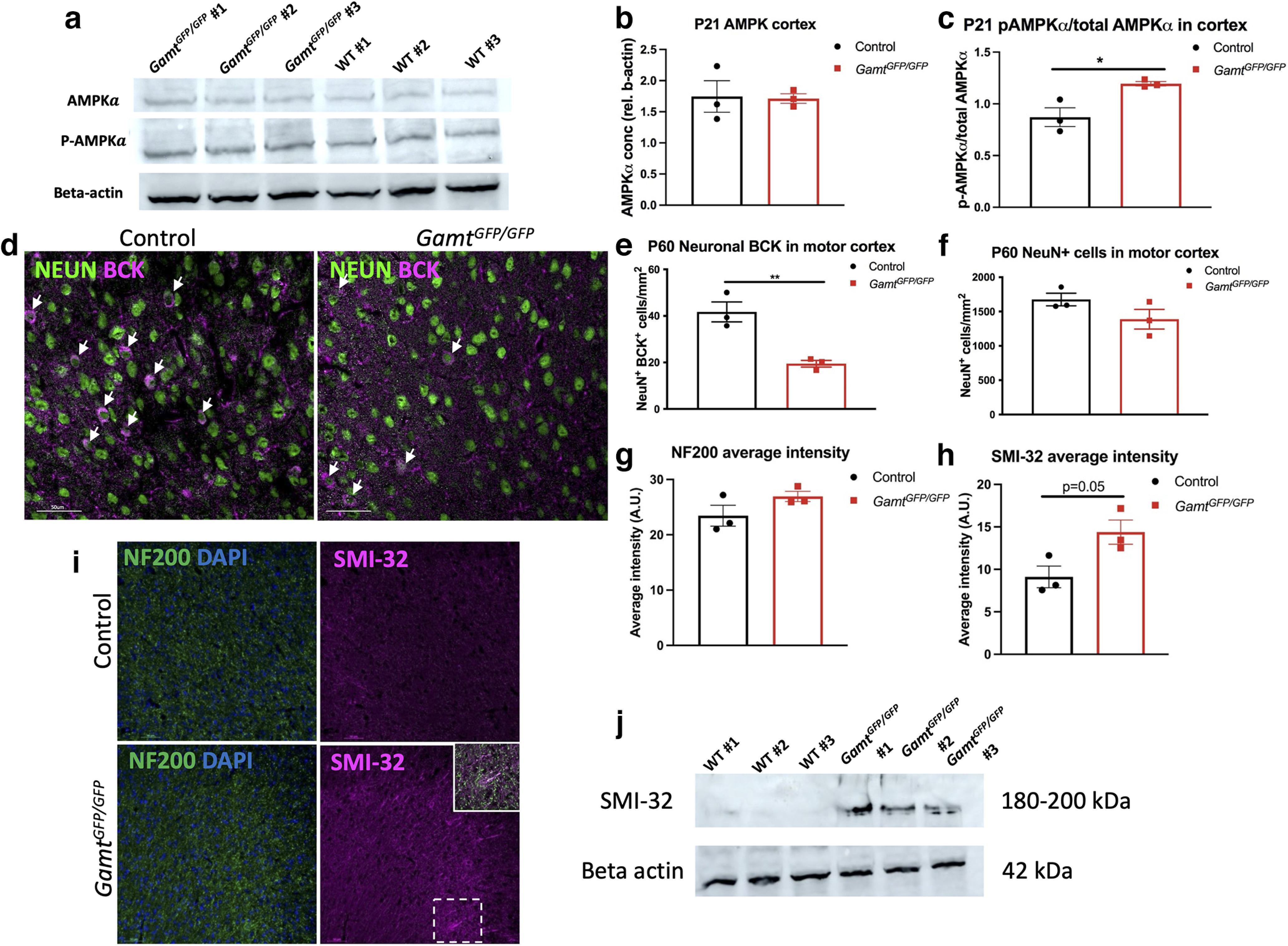
Removal of *Gamt* leads to activated AMPK signaling and reduced BCK in the cortex at P60. ***a***, Western blot of cortical lysates of AMPK and p-AMPK proteins. ***b***, Quantification showing of total AMPK protein (two-tailed *t* test; *t* = 2.884, df = 4, *p* = 0.045). ***c***, Quantification of phosphorylated AMPK (two-tailed *t* test; *t* = 6.084, df = 4, *p* = 0.0037). Western blot of cerebellar lysates for AMPK and p-AMPK proteins is shown in Extended Data Figure 5-1. ***d***, Images of BCK in neurons in the motor cortex at P60 in controls and *Gamt^GFP/GFP^*. ***e***, Quantification of BCK^+^ neuron density in the motor cortex at P60 (two-tailed *t* test; *t* = 4.912, df = 4, *p* = 0.008). ***f***, Density of neurons in the motor cortex (*p* = 0.16, not significant). Expression of BCK in the adult corpus callosum is shown in Extended Data Figure 5-2. ***g***, Intensity of neurofilament in the motor cortex at P60 (*p* = 0.2). ***h***, Intensity of nonphosphorylated neurofilament (SMI-32) (two-tailed *t* test; *t* = 2.761, df = 4, *p* = 0.05). ***i***, Images of neurofilament and nonphosphorylated neurofilament intensity in the motor cortex. ***j***, Western blot of cortical lysates of SMI-32 protein at P60. Data are mean ± SEM; *n* = 3 biological replicates. Scale bars: ***d***, ***i***, 50 µm. *p < 0.05. **p < 0.01.

Creatine is known to play an important role in ATP buffering, while also regulating cellular energetics and glucose metabolism through AMP-kinase (AMPK) phosphorylation in muscles (Hardie et al., 2012). To determine whether GAMT loss of function alters AMPK signaling in the CNS, Western blot analysis of AMPK and phospho-AMPK in cortical tissues of *Gamt^GFP/GFP^* and control mice at P21 were performed (Fig. 5*a*). We found that total AMPK protein expression was unchanged in *Gamt^GFP/GFP^* mice compared with control (Fig. 5*b*). However, the level of phospho-AMPK compared with total AMPK expression was increased in *Gamt^GFP/GFP^* mice compared with control (Fig. 5*c*). A similar increase in AMPK phosphorylation was also observed in the cerebellum of *Gamt^GFP/GFP^* compared with control (Extended Data Fig. 5-1). These findings suggest a potential shift in brain bioenergetics in the absence of endogenously synthesized creatine.

10.1523/JNEUROSCI.2120-21.2022.f5-1 Figure 5-1 Removal of *Gamt* leads to activated AMPK signaling in the cerebellum. **a**) Western blot of cerebellar lysates of total AMPK and phosphorylated AMPK. **b**) Quantification of total AMPK relative to beta actin (two-tailed t-test; t = 2.884, df = 4, p = 0.045). **c**) Quantification of phosphorylated AMPK relative to total AMPK (two-tailed t-test; t = 6.084, df = 4, p = 0.0037). Download Figure 5-1, TIF file.

10.1523/JNEUROSCI.2120-21.2022.f5-2 Figure 5-2 Brain creatine kinase colocalizes within astrocytic processes and not with oligodendrocyte lineage cells in adult corpus callosum. a) Images showing colocalization of brain creatine kinase (BCK) with GFAP^+^ astrocytic processes. b) Higher magnification of the outlined region in a) showing colocalization with astrocytes and not with OLIG2^+^ oligodendrocyte lineage cells. Scale bar is 50µm in all images. Download Figure 5-2, TIF file.

Under increased energetic demand, AMPK is known to phosphorylate BCK, an enzyme that regulates the transfer of the high-energy phosphate in phosphocreatine to ADP for local ATP regeneration (Ramírez Ríos et al., 2014). It has previously been suggested that human BCK is expressed within inhibitory neurons and astrocytes but not in oligodendrocytes (Lowe et al., 2013). We confirmed that BCK is expressed in a subpopulation of NeuN^+^ neurons and predominantly expressed in GFAP^+^ astrocytic processes but does not colocalize with OLIG2^+^ oligodendrocyte lineage cells (Fig. 5*d*; Extended Data Fig. 5-2). To determine whether loss of GAMT affected BCK expression, immunostaining analysis of *Gamt^GFP/GFP^* and control mice at P60 was performed (Fig. 5*d*). We found that BCK expression was significantly reduced in the cortex of *Gamt^GFP/GFP^* mice compared with controls, and particularly in NeuN^+^ neurons (Fig. 5*e*). However, this reduction was not associated with neuronal loss since we found no difference in the overall density of NeuN^+^ neurons between *Gamt^GFP/GFP^* and control mice (Fig. 5*f*). To determine whether GAMT deletion affected axonal integrity in the adult CNS, analysis of nonphosphorylated neurofilament (SMI-32) was performed. We observed an increase in SMI-32 staining in the cortex of *Gamt^GFP/GFP^* mice compared with control (Fig. 5*h*,*i*), but no difference in total neurofilament staining (Fig. 5*g*). Additionally, Western blot analysis of cortex lysates also showed increased SMI-32 levels in *Gamt^GFP/GFP^* mice compared with control (Fig. 5*j*). These results suggest that GAMT deletion resulted in altered energetics and impaired axonal integrity in the adult CNS.

### Conditional deletion of *Gamt* in oligodendrocyte lineage cells leads to reduced oligodendrocytes and inefficient remyelination after demyelinating injury

**Figure 6.**
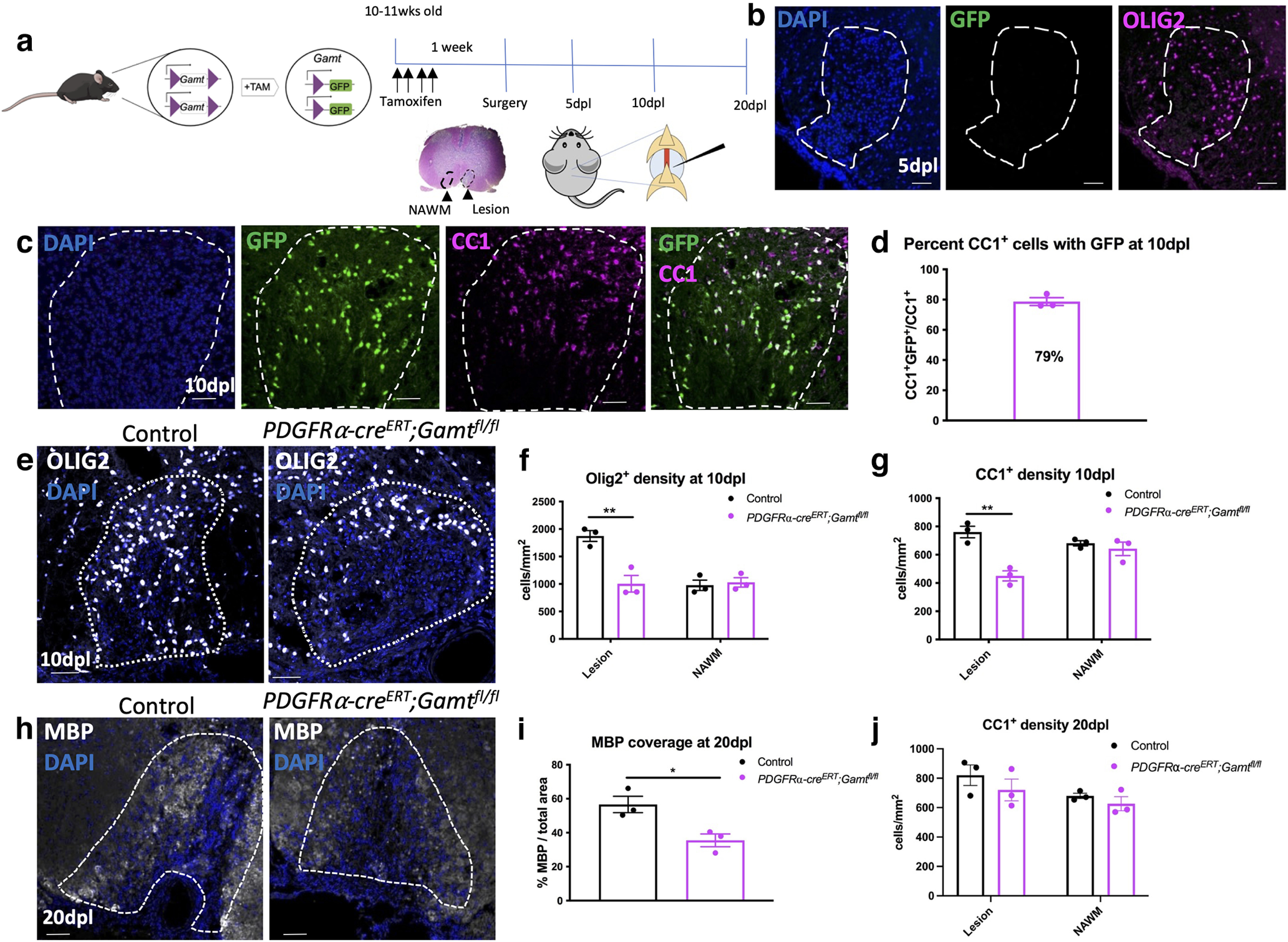
Deletion of *Gamt* from oligodendrocyte lineage cells leads to a reduction in mature oligodendrocytes and myelin coverage after demyelinating injury. ***a***, Diagram of transgenic mouse model showing removal of *Gamt* from OL lineage cells after 4 d of intraperitoneal tamoxifen injections. ***b***, No GFP expression at 5 dpl. ***c***, Colocalization of GFP^+^CC1^+^ oligodendrocytes in lesions at 10 dpl. ***d***, Quantification showing percentage of CC1^+^ oligodendrocytes with GFP expression in lesions at 10 dpl. ***e***, Images of OLIG2^+^ oligodendrocyte lineage cells in the lesion. ***f***, Quantification of OLIG2^+^ cell density in lesion and adjacent normal appearing white matter (NAWM) of OL *Gamt* iKO and control mice (two-way ANOVA with Tukey's multiple comparisons; *F*_(1,8)_ = 13.76, df = 11, *p* = 0.007). ***g***, Quantification of CC1^+^ oligodendrocytes at 10 dpl (two-way ANOVA with Tukey's multiple comparisons; *F*_(1,8)_ = 0.0016, df = 1, *p* = 0.0016). ***h***, MBP staining at 20 dpl. ***i***, Quantification of MBP coverage at 20 dpl (two-tailed *t* test; *t* = 3.449, df = 4, *p* = 0.026). ***j***, Quantification of CC1^+^ mature oligodendrocyte density at 20 dpl (*p* = 0.69, not significant) Data are mean ± SEM; *n* = 3 biological replicates. Scale bars: ***b***, ***c***, ***e***, ***h***, 50 µm. *p < 0.05. **p < 0.01.

We next determined whether *Gamt* expression in oligodendrocytes is required for remyelination. To this end, we crossed the *Gamt^fl/fl^* line with the tamoxifen-inducible *PDGFR*ɑ-*Cre^ERT^* KO mouse line, and generated *PDGFR*ɑ-*Cre^ERT^*;*Gamt^fl/fl^* iKO mice to allow the conditional deletion of *Gamt* in oligodendrocyte lineage cells (Fig. 6*a*). To ensure dietary creatine did not influence oligodendrocyte lineage cell function during remyelination, all mice were fed a creatine-deficient diet. For oligodendrocyte lineage cell *Gamt* deletion, tamoxifen was injected intraperitoneally into mice at 10-11 weeks of age for 4 consecutive days before demyelination, and focal experimental demyelination was then performed by lysolecithin injection into the mouse spinal cord ventral white matter. This protocol for tracking remyelination in the spinal cord has been well characterized by our laboratory (Chamberlain et al., 2017). Sibling tamoxifen-injected *Gamt^fl/fl^* mice without the *PDGFR*ɑ-*Cre^ERT^* allele were used as controls. To confirm *Gamt* deletion, we examined the expression of GFP in the lesioned spinal cord of *PDGFR*ɑ-*Cre^ERT^*;*Gamt^fl/fl^* iKO and control mice. At 5 d post lesion (dpl), when OPCs are expected to migrate to and proliferate in lesions, we did not observe any GFP expression in or outside of the lesions of *PDGFR*ɑ-*Cre^ERT^*;*Gamt^fl/fl^* iKO or control mice (Fig. 6*b*), suggesting that *Gamt* is not expressed early in remyelination. The lack of GFP expression also suggested that no mature oligodendrocytes that survived the lesion expressed GFP. However, at ∼10 dpl, when recruited OPCs are expected to have begun to differentiate into oligodendrocytes, we detected GFP expression only in CC1^+^ oligodendrocytes in lesions and barely outside of lesions (Fig. 6*c*). Quantification of GFP-labeled oligodendrocytes showed ∼79% of mature oligodendrocytes expressed GFP in lesions (Fig. 6*d*), suggesting that GFP^+^ oligodendrocytes were derived from OPCs that have migrated into the lesion during remyelination. These results demonstrate the inducible GFP tagging approach using the *PDGFR*ɑ-*Cre^ERT^*;*Gamt^fl/fl^* line allows the identification of *Gamt* deleted oligodendrocyte lineage cells after demyelinating injury, and the tracking of newly regenerated oligodendrocytes in lesions.

To determine whether tamoxifen-induced *Gamt* deletion in *PDGFR*ɑ-*Cre^ERT^*;*Gamt^fl/fl^* iKO mice affected oligodendrocyte lineage cell progression in lesions, immunostaining analysis for oligodendrocyte lineage cell markers was performed. We detected a significant reduction of OLIG2^+^ oligodendrocyte lineage cells and CC1^+^ mature oligodendrocytes in lesions in the *PDGFR*ɑ-*Cre^ERT^*;*Gamt^fl/fl^* iKO compared with control at 10 dpl (Fig. 6*e–g*). Moreover, MBP coverage in the lesion, which is an indicator of remyelination, was significantly reduced at 20 dpl (Fig. 6*h*,*i*). However, by 20 dpl, there was no longer a difference in total mature oligodendrocytes in the lesion (Fig. 6*j*). Although MBP coverage was still reduced in the *PDGFR*ɑ-*Cre^ERT^*;*Gamt^fl/fl^* iKO after 20 dpl, the increase in oligodendrocyte density to comparable levels as controls suggests that the loss of *Gamt* in oligodendrocyte lineage led to a transient delay in oligodendrocyte maturation following injury. These results suggest that endogenous creatine synthesis in oligodendrocytes influences the timing of oligodendrocyte maturation/remyelination but is not required for these processes.

### Creatine or cyclocreatine supplemented diets increase mature oligodendrocytes and enhance remyelination after cuprizone-mediated demyelination

**Figure 7.**
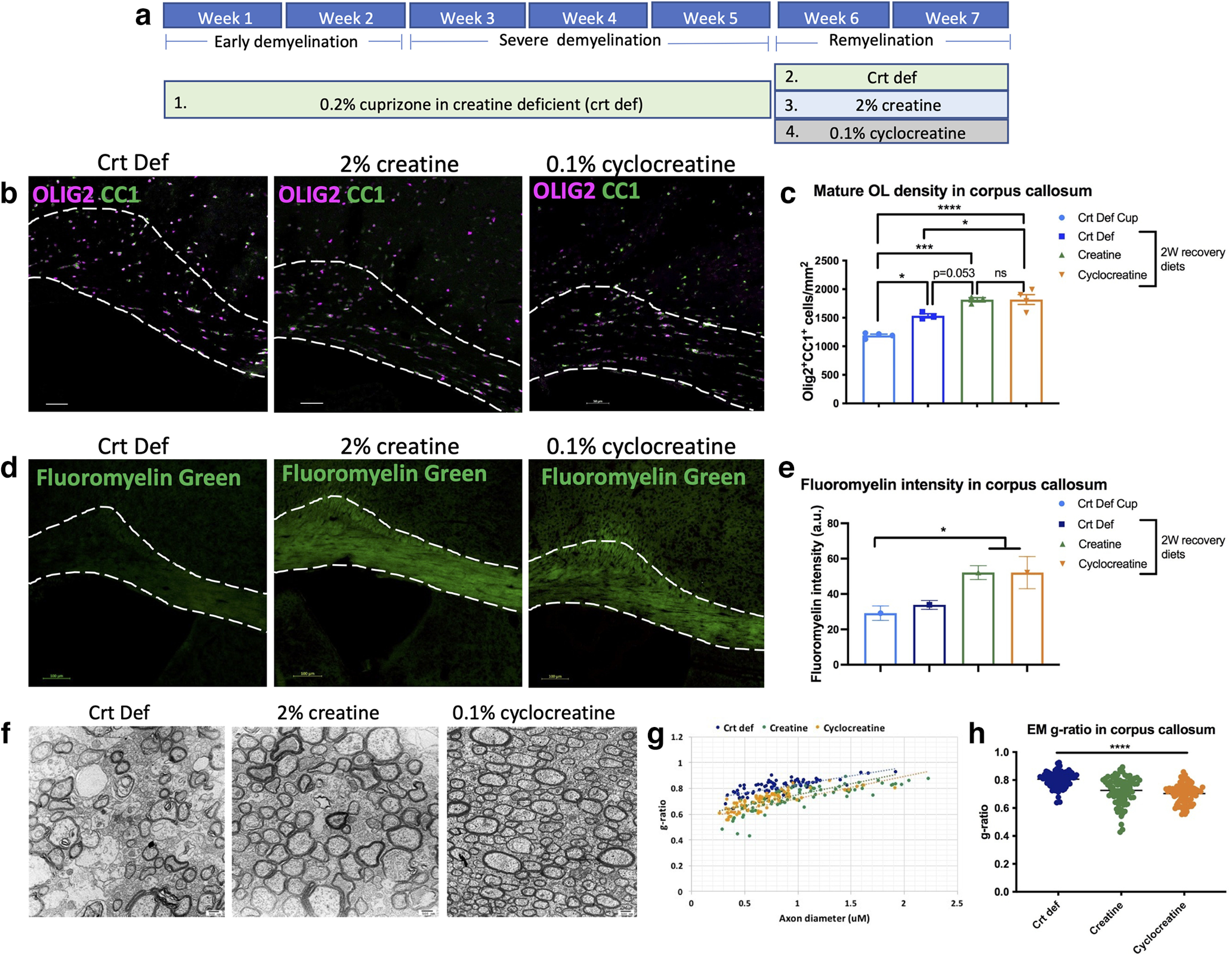
Creatine and cyclocreatine supplemented recovery diets lead to increased mature oligodendrocytes and enhanced remyelination. ***a***, Outline of diet for each mouse group for the cuprizone demyelination experiment. ***b***, Images of OLIG2^+^CC1^+^ oligodendrocytes in corpus callosum after 2 weeks of recovery diet (creatine-deficient, 2% creatine and 0.1% cyclocreatine). ***c***, Density of mature oligodendrocytes (one-way ANOVA with Sidak's multiple comparisons; *F*_(3,10)_ = 29.58, df = 13, *p* < 0.0001). ***d***, Images of fluoromyelin in corpus callosum after various recovery diets. ***e***, Quantification of fluoromyelin intensity in the different groups (one-way ANOVA with Dunnett's multiple comparisons; *F*_(3,11)_ = 4.279, df = 14, *p* = 0.03). ***f***, EM images of axons in cross sections of corpus callosum. Original magnification × 5000. ***g***, ***h***, g-ratio analysis of creatine and cyclocreatine groups (one-way ANOVA with Tukey's multiple comparisons; *F*_(2,237)_ = 40.28, df = 239, *p* < 0.0001). Data are mean ± SEM; *n* = 3 or 4 biological replicates in ***a–d***. Representative EM analysis in ***f–h*** from *n* = 1, 80 axons per animal. Scale bars: ***b***, 50 µm; ***d***, 100 µm; ***f***, 500 nm. *p < 0.05. **p < 0.01. ***p < 0.001. ****p < 0.0001.

To determine whether creatine gain of function affects remyelination, cuprizone demyelination was performed on WT mice fed with a creatine-deficient diet for 5 weeks (demyelination period; Group 1), followed by a switch to a diet without cuprizone containing either 2% creatine or 0.1% cyclocreatine, a planar creatine analog with greater brain penetrance, for 2 weeks (recovery period; Fig. 7*a*; Groups 2-4). Remyelination in all three recovery groups was compared with the creatine-deficient cuprizone group (Fig. 7*a*; Group 1). The creatine-deficient cuprizone group was killed immediately following 5 weeks of cuprizone. The cuprizone demyelination model was used because it allowed us to monitor remyelination efficiency in the corpus callosum under long-term creatine or cyclocreatine treatment (Torkildsen et al., 2008). We found that both creatine and cyclocreatine treatment increased the number of OLIG2^+^CC1^+^ oligodendrocytes (Fig. 7*b*,*c*), and significantly greater fluoromyelin staining in the corpus callosum compared with control after 2 weeks of recovery diet (Fig. 7*d*,*e*). Moreover, EM analysis revealed that creatine and cyclocreatine diets increased the extent of remyelinated axons compared with control, as well as lower g-ratios (Fig. 7*f–h*). These results suggest that dietary creatine significantly enhanced the rate of CNS remyelination in mice.

## Discussion

### Creatine synthesis through *Gamt* supports oligodendrocyte maturation and survival during developmental myelination

Clinical symptoms in CCDS suggest that the CNS is particularly vulnerable to creatine deficiency (Giusti et al., 2019). We have performed MRS analysis and showed that cerebral creatine levels are dependent on *Gamt* expression. Moreover, we have now generated a new transgenic mouse model that enables GFP tagging of cells normally displaying creatine synthesis and investigation of the effect of endogenous creatine loss on cellular function. The ability to perform cell-specific deletion of *Gamt* may aid in understanding the etiology behind the development of the intellectual disabilities, seizures, and behavior disorders observed in CCDS patients. We found that *Gamt* is not expressed in OPCs but is expressed in a small portion of early myelinating oligodendrocytes and highly expressed in mature oligodendrocytes, as was observed in previous studies (Tachikawa et al., 2018; Baker et al., 2021), and suggest that creatine synthesis may support the energetic demand required for developmental myelination. Our *Gamt^GFP/GFP^* KO model showed significant reductions in mature oligodendrocytes and myelin proteins at P14 and P21 compared with control, and coincided with increased cell death in early myelinating oligodendrocytes. However, the reduction of oligodendrocytes was no longer observed P60, suggesting that endogenous creatine synthesis is not required for myelination in mice but may be necessary to ensure that oligodendrocyte maturation and myelination occur in a timely manner in the postnatal brain (Hughes and Stockton, 2021). It is known that alterations in the CNS during critical windows of development can have long-lasting impacts on brain function and associated behavior (Marín, 2016). However, the lack of any overt behavioral perturbations in our mouse model may be attributed to the difference in neocortical development and white matter volume between humans and rodents.

We found that mice lacking *Gamt* expression displayed increased AMPK signaling, and that neurons in the adult CNS exhibited decreased BCK expression and increased SMI-32 staining in the cortex at P60, suggesting that creatine deficiency results in altered neuronal bioenergetics and decreased neuronal integrity (Yandamuri and Lane, 2016). It remains unknown whether oligodendrocyte-derived creatine regulates oligodendrocyte function cell-autonomously, or can be distributed to other cell types in the brain, thereby affecting neighboring cells non–cell-autonomously (Fünfschilling et al., 2012; Lee et al., 2012). Unfortunately, attempts to delete *Gamt* in oligodendrocyte lineage cells in newborn mice through tamoxifen injections have not been successful. Therefore, it remains to be determined whether the observed dysregulation in neuronal bioenergetics from *Gamt* deletion occurred from (1) a reduced local supply of creatine from oligodendrocytes, (2) oligodendrocyte dysfunction or loss during development, or (3) a potential role of *Gamt* in neural stem/precursor cell differentiation or function early in development. Importantly, previous studies of *Gamt* expression in CNS development using *in situ* hybridization suggest that *Gamt* may be expressed in low levels in neurons and astrocytes in addition to oligodendrocyte expression (Braissant et al., 2001). Although we did not observe any GFP expression in neurons in our study, we cannot completely rule out low levels of *Gamt* expression in neurons that were undetectable under standard confocal fluorescence microscopy, and therefore cannot be certain any neuronal changes observed resulted directly from the disruption of creatine synthesis in oligodendrocytes.

### Endogenous creatine synthesis and dietary creatine supplementation support remyelination

We found that creatine synthesis in oligodendrocytes is important for the remyelination process. Interestingly, following lysolecithin demyelination of tamoxifen-injected *PDGFR*ɑ-*Cre^ERT^*;*Gamt^fl/fl^* iKO mice, we saw no expression of the GFP reporter at 5 dpl and only in mature oligodendrocytes in the lesion at 10 dpl, suggesting that the GFP-tagged oligodendrocytes in lesions are newly regenerated oligodendrocytes involved in remyelination. The inducible GFP tagging approach may be useful for the identification and tracking of newly regenerated oligodendrocytes and could be incorporated into other animal models of demyelination or experimental injury. Since half of daily creatine level in humans comes from diet, we investigated whether dietary creatine or cyclocreatine, a lipophilic analog with greater brain penetrance, affects the efficiency of remyelination after cuprizone demyelination. We found that both creatine and cyclocreatine increased the number of oligodendrocytes and improved remyelination, but there was no significant difference between the two diets used. Increasing the cyclocreatine dose or limiting the length of recovery diet may allow us to see greater differences between the diets. Overall, this experiment suggests that creatine supplementation can improve the rate of remyelination.

In conclusion, we found that mature oligodendrocytes actively synthesize creatine during myelination, and that impaired creatine synthesis through *Gamt* loss results in delayed myelination during development and after injury. Moreover, we found that dietary creatine enhances CNS remyelination. The lack of endogenously synthesized creatine may affect neuronal energetics and function, but we cannot exclude the possibility that altered neuronal function occurred through GAMT loss in cells other than oligodendrocytes in the adult CNS. These results suggest oligodendrocyte dysfunction might be a contributor to the CNS pathophysiology observed under creatine deficiency conditions, such as CCDS.
