# Supplementary figures and images for: Cerebral Creatine Deficiency Affects the Timing of Oligodendrocyte Myelination
Source: J Neurosci. 2023 Feb 15;43(7):1143–53. doi: 10.1523/JNEUROSCI.2120-21.2022 (PMC9962777; doi:10.1523/JNEUROSCI.2120-21.2022)

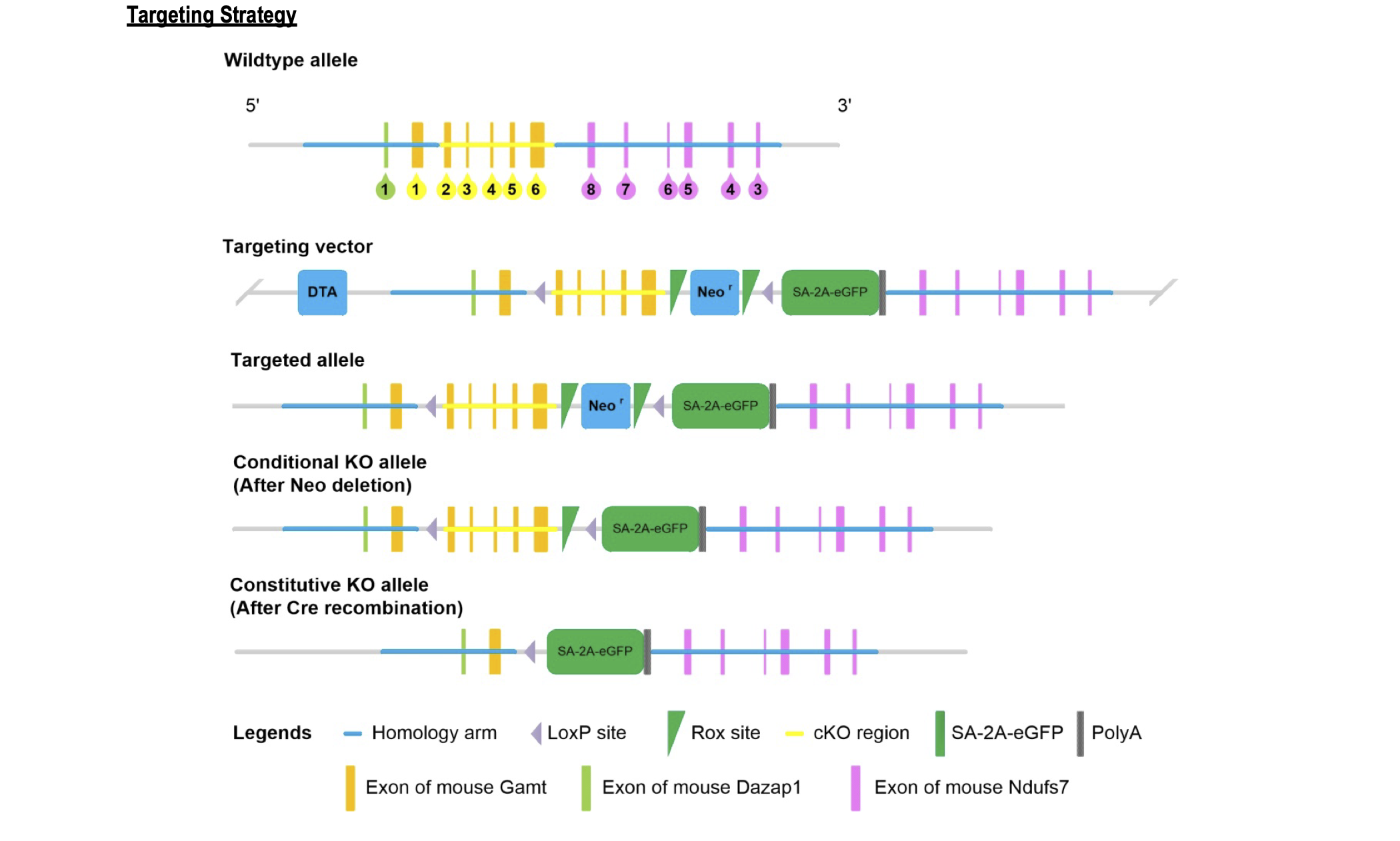

Supplement: Figure 2-1 — Overview of targeting strategy of transgenic mouse line. The line was engineered and generated by Cyagen biosciences A linearized vector was generated to the Gamt gene in C57BL/6 mice on chromosome ten and delivered to embryonic stem cells via electroporation. The targeted allele has loxP sites (purple) flanking exons 2-6 of the Gamt gene (yellow). The green Rox site prevents expression of enhanced GFP cassette but upon Cre recombination, Gamt along with the Rox site are removed and GFP is expressed. Download Figure 2-1, TIF file. [file ns-JN-RM-2120-21-s01.tif]

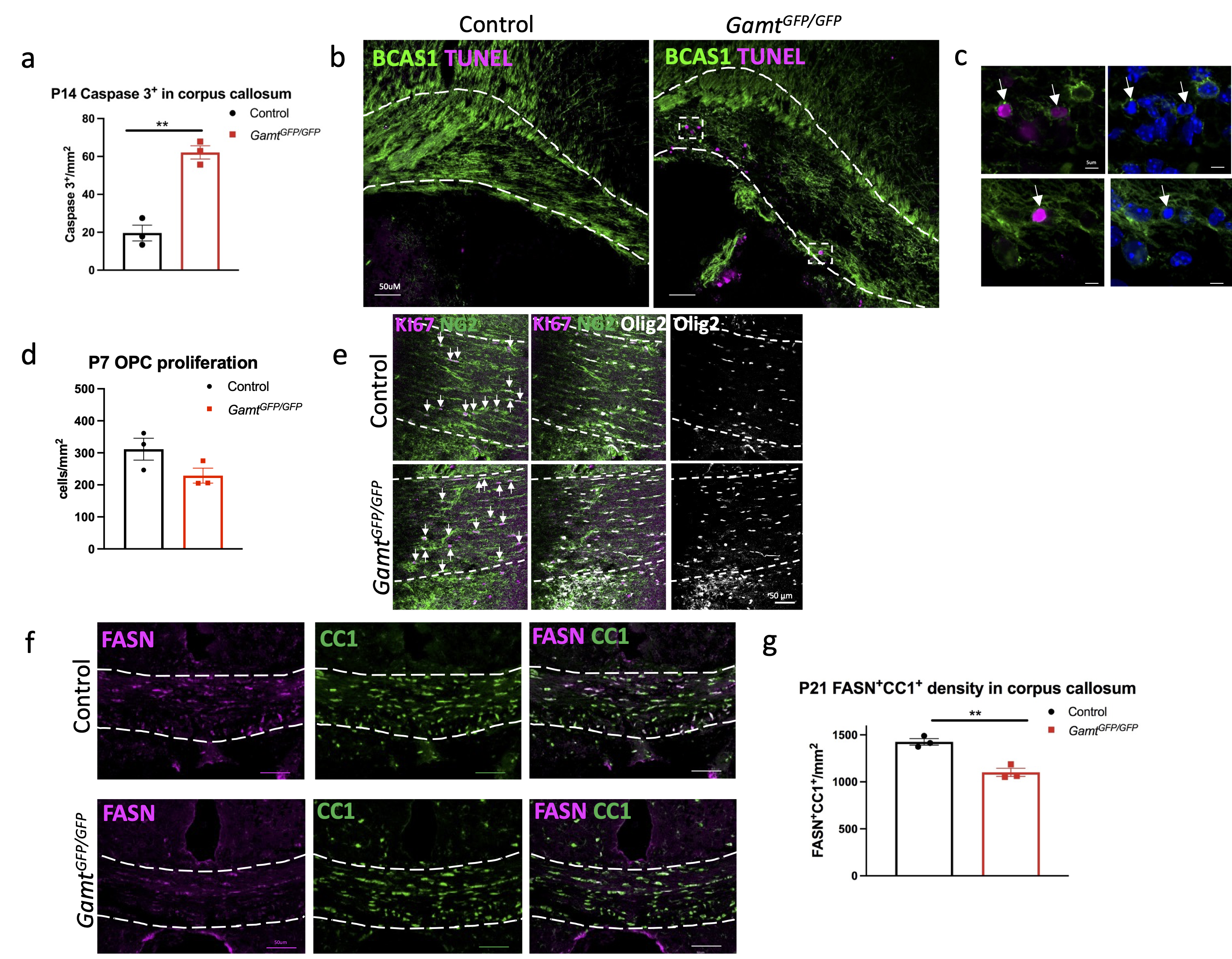

Supplement: Figure 4-1 — Removal of GAMT leads to increased cell death and reduced fatty acid synthase in the corpus callosum. a) Quantification of caspase-3+ cell density at P14 (two-tailed t-test; t = 7.81, df = 4, p = 0.0015). b) Images of BCAS1+TUNEL+ dying early myelinating cells. c) Inserts showing colocalization of BCAS1+TUNEL+ in c. d) Quantification of OPC proliferation at P7 (two-tailed t-test; t = 2.005, p = 0.1155). e) Images of P7 OPC proliferation. f) Images of FASN+CC1+ cells in the corpus callosum at P21. g) Quantification of FASN+CC1+ cells (two-tailed t-test; t = 5.88, df = 4, p = 0.0042). Data are mean ± SEM with n = 3 biological replicates. Scare bar is 50µm in b and d. **p < 0.01. Download Figure 4-1, TIF file. [file ns-JN-RM-2120-21-s02.tif]

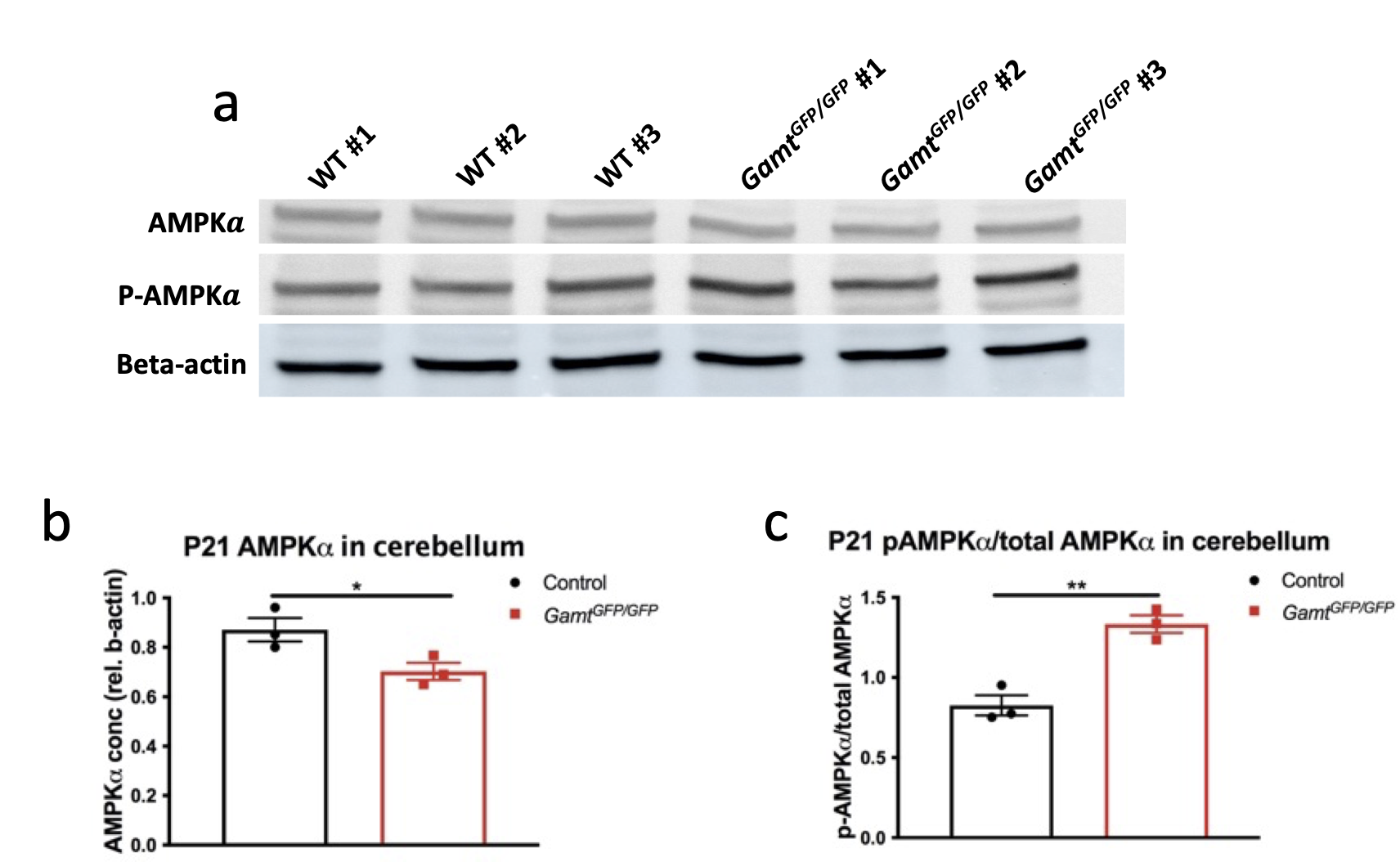

Supplement: Figure 5-1 — Removal of Gamt leads to activated AMPK signaling in the cerebellum. a) Western blot of cerebellar lysates of total AMPK and phosphorylated AMPK. b) Quantification of total AMPK relative to beta actin (two-tailed t-test; t = 2.884, df = 4, p = 0.045). c) Quantification of phosphorylated AMPK relative to total AMPK (two-tailed t-test; t = 6.084, df = 4, p = 0.0037). Download Figure 5-1, TIF file. [file ns-JN-RM-2120-21-s03.tif]

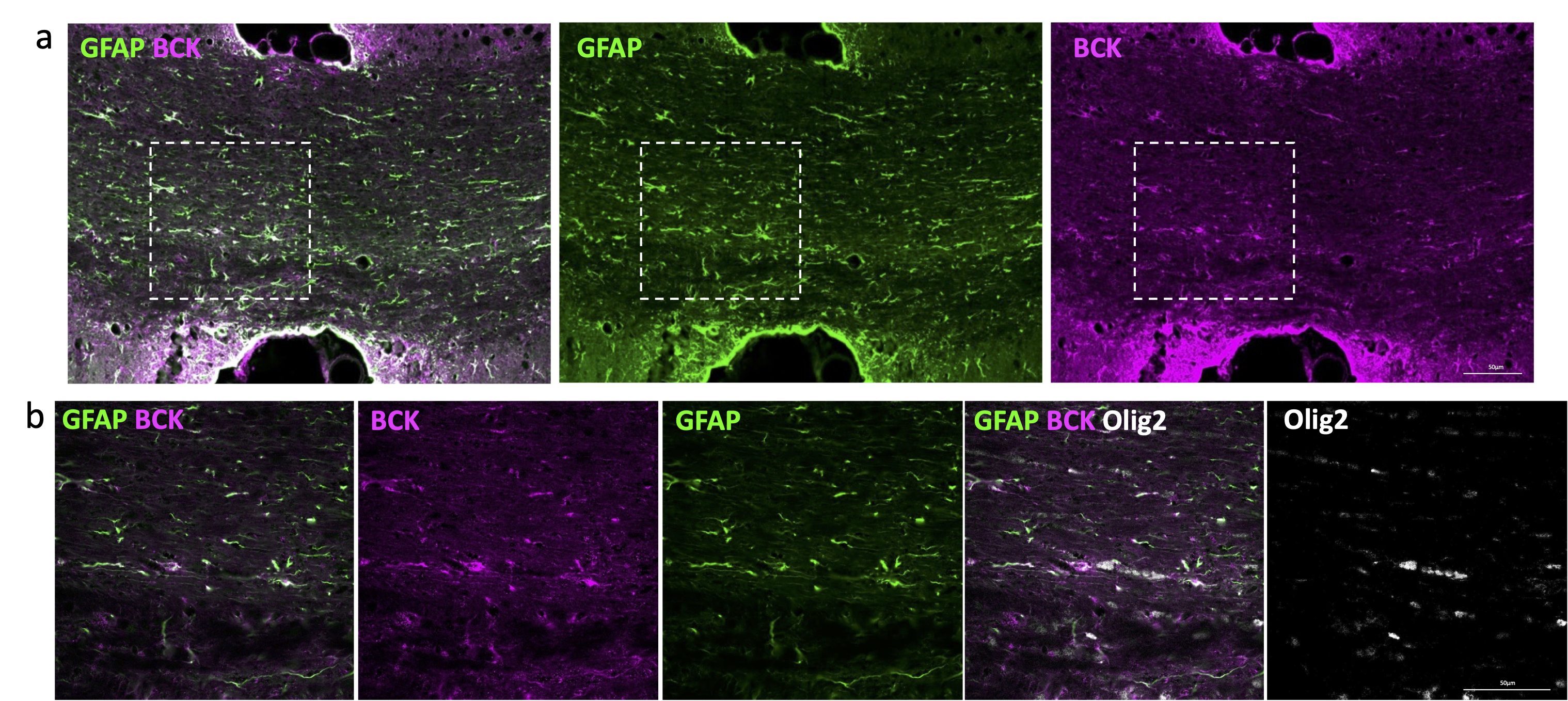

Supplement: Figure 5-2 — Brain creatine kinase colocalizes within astrocytic processes and not with oligodendrocyte lineage cells in adult corpus callosum. a) Images showing colocalization of brain creatine kinase (BCK) with GFAP+ astrocytic processes. b) Higher magnification of the outlined region in a) showing colocalization with astrocytes and not with OLIG2+ oligodendrocyte lineage cells. Scale bar is 50µm in all images. Download Figure 5-2, TIF file. [file ns-JN-RM-2120-21-s04.tif]
